# Systemically Delivered Magnetic Hyperthermia for Prostate Cancer Treatment

**DOI:** 10.3390/pharmaceutics12111020

**Published:** 2020-10-25

**Authors:** Hassan A. Albarqi, Ananiya A. Demessie, Fahad Y. Sabei, Abraham S. Moses, Mikkel N. Hansen, Pallavi Dhagat, Olena R. Taratula, Oleh Taratula

**Affiliations:** 1 Department of Pharmaceutical Sciences, College of Pharmacy, Oregon State University, Portland, OR 97201, USA; haalbarqi@nu.edu.sa (H.A.A.); demessia@oregonstate.edu (A.A.D.); sabeif@oregonstate.edu (F.Y.S.); mosesab@oregonstate.edu (A.S.M.); 2 Department of Pharmaceutics, College of Pharmacy, Najran University, Najran 61441, Saudi Arabia; 3 Department of Pharmaceutics, College of Pharmacy, Jazan University, Jazan 88723, Saudi Arabia; 4 School of Electrical Engineering and Computer Science, Oregon State University, Corvallis, OR 97331, USA; hansemik@oregonstate.edu (M.N.H.); dhagat@oregonstate.edu (P.D.)

**Keywords:** nanoparticle, nanocluster, magnetic hyperthermia, alternating magnetic field, prostate cancer

## Abstract

Herein, we report a novel therapy for prostate cancer based on systemically delivered magnetic hyperthermia. Conventional magnetic hyperthermia is a form of thermal therapy where magnetic nanoparticles delivered to cancer sites via intratumoral administration produce heat in the presence of an alternating magnetic field (AMF). To employ this therapy for prostate cancer tumors that are challenging to inject intratumorally, we designed novel nanoclusters with enhanced heating efficiency that reach prostate cancer tumors after systemic administration and generate desirable intratumoral temperatures upon exposure to an AMF. Our nanoclusters are based on hydrophobic iron oxide nanoparticles doped with zinc and manganese. To overcome the challenges associated with the poor water solubility of the synthesized nanoparticles, the solvent evaporation approach was employed to encapsulate and cluster them within the hydrophobic core of PEG-PCL (methoxy poly(ethylene glycol)-*b*-poly(*ε*-caprolactone))-based polymeric nanoparticles. Animal studies demonstrated that, following intravenous injection into mice bearing prostate cancer grafts, the nanoclusters efficiently accumulated in cancer tumors within several hours and increased the intratumoral temperature above 42 °C upon exposure to an AMF. Finally, the systemically delivered magnetic hyperthermia significantly inhibited prostate cancer growth and did not exhibit any signs of toxicity.

## 1. Introduction

The high mortality rate among prostate cancer patients is attributed to the fact that there is no effective cure once the disease has spread beyond the prostate. Hormone therapy can initially slow the growth of prostate cancer that has spread, but many patients eventually develop hormone-refractory prostate cancer [1,2]. Chemotherapy, currently being used to treat hormone-refractory prostate cancer, has limited efficacy and causes side effects on healthy organs [2]. Therefore, there is a critical need to develop novel therapies for advanced prostate cancer that will efficiently kill malignant prostate cells and minimize severe side effects.

Magnetic hyperthermia is a form of thermal therapy where magnetic nanoparticles produce high temperatures at cancer sites in the presence of a noninvasive alternating magnetic field (AMF). This feature allows a remote elevation of the intratumoral temperature while minimizing the heating of healthy tissue [3,4]. Magnetic nanoparticles can be used to generate both mild hyperthermia (42–46 °C) and high-temperature hyperthermia (thermal ablation, >46 °C) in cancer tissues in order to destroy tumors, inhibit their growth, or enhance the sensitivity of tumors to other therapies [5,6]. Many preclinical studies suggest that magnetic hyperthermia has significant potential to kill cancer cells directly or enhance their susceptibility to radiation and chemotherapy [7,8]. Recent reports also suggest that magnetic hyperthermia elicits anti-tumor immune responses and can work synergistically with immunotherapy for cancer treatment [9,10]. Importantly, the feasibility of magnetic hyperthermia has already been validated in clinical studies on patients with prostate cancer [11,12,13]. Various clinical studies have also verified that the addition of magnetic hyperthermia to radiation and chemotherapy enhances tumor control and patient survival rates [14,15]. Conventional magnetic hyperthermia, however, is restricted to the treatment of accessible tumors because therapeutically relevant temperatures (>40 °C) are only reached following the intratumoral administration of magnetic nanoparticles [16,17]. This is due to the relatively low tumor accumulation and heating efficiency of conventional iron oxide nanoparticles following systemic delivery. Therefore, systemically delivered magnetic hyperthermia has not yet been explored for the treatment of various types of cancer, including prostate cancer.

We recently reported novel nanoclusters of hexagon-shaped iron oxide nanoparticles that can elevate temperatures in ovarian cancer grafts over 42 °C after the systemic administration of a clinically relevant dose (<10 mg Fe/kg) and exposure to a safe AMF [16]. To develop iron oxide nanoparticles with high heating efficiency, their cores were doped with manganese and cobalt. The experimental results confirmed that negligible leaching of cobalt from the nanoparticles was detected after incubation in serum at 37 °C for seven days and that nanoclusters are not toxic. Nevertheless, we realize that cobalt-containing nanoclusters might have limited translational potential due to theoretical concerns about cobalt leaching from the nanoclusters, resulting in toxicity [18].

The main aim of this work was the development of novel cobalt-free nanoclusters doped with zinc (Zn) and manganese (Mn) that possessed a heating efficiency similar to that of nanoclusters made with cobalt but without the putative toxicity. Among metal dopants, Mn and Zn are considered to be the safest magnetic metals to which the human body can be exposed at a low dosage and the softest ferrite materials that have high saturation magnetization, resulting in the enhanced heating efficiency of nanoparticles [19]. Furthermore, in this report, we demonstrate, for the first time, the feasibility of systemically delivered magnetic hyperthermia for the treatment of prostate cancer tumors.

## 2. Materials and Methods

### 2.1. Materials

Iron(III) acetylacetonate (Fe(acac)_3_) was obtained from Acros Organics (Fair Lawn, NJ, USA). Zinc chloride anhydrous (ZnCl_2_) and oleylamine were purchased from Sigma-Aldrich (St. Louis, MO, USA). Oleic acid and manganese(II) chloride tetrahydrate (MnCl_2_·4H_2_O) were obtained from Alfa Aesar (Ward Hill, MA, USA). *n*-Octyl ether was purchased from Tokyo Chemical Industry Co. (Tokyo, Japan). Methoxy poly(ethylene glycol)-*b*-poly(ε-caprolactone) (PEG-PCL, MW: 5 k–10 k) was obtained from Advanced Polymer Materials Inc. (Montreal, Québec, Canada). Silicon 2,3-naphthalocyanine bis(trihexylsilyloxide) (SiNc) was purchased from Sigma-Aldrich (Milwaukee, WI, USA). All other chemicals and supplies were obtained from VWR International (Radnor, PA, USA).

### 2.2. Synthesis of Zn- and Mn-Doped Iron Oxide Nanoparticles

Iron oxide nanoparticles doped with Zn and Mn (ZnMn-IONPs) were prepared according to a recently published protocol with some modification [16,20,21]. ZnCl_2_ (2 mmol), MnCl_2_·4H_2_O (3 mmol) and Fe(acac)_3_ (5 mmol) were combined in a 250 mL flask with oleic acid (6 mmol), oleylamine (6 mmol) and *n*-octyl ether (25 mL). The obtained mixture was heated to 300 °C and stirred under nitrogen flow. After 1 h at 300 °C, the black mixture was precipitated, washed three times with ethanol (30 mL) and centrifuged for 30 min. The precipitate was re-dispersed in hexane. The obtained ZnMn-IONPs were dried at 70 °C for 12 h.

### 2.3. Characterization of Nanoparticles

The surface morphology and particle size of the ZnMn-IONPs were evaluated with a FEI Tecnai™ Spirit transmission electron microscopy (TEM) system [16]. To characterize the chemical composition of the nanoparticles, a FEI G2 80–300 kV Titan (S) TEM equipped with a spherical aberration image corrector was used. The element composition was analyzed by energy dispersive X-ray spectroscopy (EDX). EDX analysis was employed to confirm the presence and the location of elements (Fe, Zn and Mn) in a single nanoparticle [16]. The saturation magnetization and heating efficiency of the nanoparticles were evaluated according to our previously described procedures [16].

### 2.4. Preparation of Nanoclusters

The solvent evaporation method was used to prepare nanoclusters of ZnMn-IONPs (ZnMn-IONCs) as previously described [16]. Briefly, ZnMn-IONPs (8 mg of Fe/mL in tetrahydrofuran (THF)) and PEG–PCL (10 mg/mL in THF) were added to 4 mL of THF and were mixed for 20 min. Then, a 5% dextrose solution in water (6 mL) was added to the mixture of ZnMn-IONPs and PEG–PCL and mixed for 20 min. The THF was then evaporated under vacuum. The ZnMn-IONCs were separated from the excess of empty PEG-PCL nanoparticles using a magnet (SuperMag Separator, Ocean NanoTech, San Diego, CA, USA). The obtained ZnMn-IONCs were re-dispersed in 5% dextrose, filtered through a 0.2 µm filter to remove any possible aggregate, analyzed in terms of loading efficiency by following our previously reported procedure [16], and stored at 4 °C.

### 2.5. Preparation of SiNc-Loaded Nanoclusters

Briefly, ZnMn-IONPs (8 mg of Fe/mL in THF) and PEG–PCL (10 mg/mL in THF) were mixed for 10 min. Then, silicon naphthalocyanine (SiNc, 0.3 mg/mL in THF) was added to the mixture, and the total volume was adjusted with THF to 6 mL, followed by stirring for 10 min. Then, a 5% dextrose solution (6 mL) was added, and the THF was evaporated under vacuum as described above. The obtained SiNc-loaded ZnMn-IONCs were filtered through a 0.2 µm filter to remove any possible aggregate.

### 2.6. Characterization of Nanoclusters

The hydrodynamic diameter and zeta potential of the prepared ZnMn-IONCs were determined by dynamic light scattering (DLS, ZetaSizer NanoSeries, Malvern, Worcestershire, UK) [16,22,23]. Transmission electron microscopy (TEM) was used to determine the morphology of the nanoclusters [16]. The heating efficiency of the nanoclusters was evaluated, as previously reported [16].

### 2.7. In Vitro Studies

The DU145 human prostate carcinoma cell line and HEK-293 human embryonic kidney cell line were obtained from Developmental Therapeutics Core (Northwestern University, Evanston, IL, USA) and ATCC (Manassas, VA, USA), respectively. To assess the cytotoxicity of the nanoclusters, cells were seeded in a 96-well plate (1 × 10^4^ cells/well) and incubated in RPMI for 24 h. The medium was replaced with fresh medium containing either previously reported nanoclusters of non-doped iron oxide nanoparticles (IONCs) [16] or ZnMn-IONCs at various concentrations (50–200 μg of Fe/mL) and incubated for 24 h. Afterward, the cells were rinsed with DPBS three times, and a modified Calcein AM assay was used to evaluate cell viability [22,24].

To assess cellular internalization, DU145 cancer cells (1 × 10^5^ cells/well) were plated in 6-well plates, followed by 24 h of incubation with SiNc-ZnMn-IONCs (50 μg of Fe/mL). The cells were washed with DPBS three times and imaged using an EVOS FL Cell Imaging System [25].

To evaluate the nanocluster-mediated hyperthermia in vitro, we followed our previously published protocol [16,26]. Briefly, DU145 cells were incubated with 50 μg of Fe/mL ZnMn-IONCs and IONCs for 24 h. Then, the cells were washed three times with DPBS, detached and resuspended in RPMI. The cells (5 × 10^6^) were resuspended in 0.1 mL of cell culture medium and centrifuged to form cell pellets. The formed pellets were then placed in a coil and exposed to an AMF (420 kHz, 26.9 kA/m) for 30 min. Temperature changes in the cell pellets were measured using a fiber-optic probe. Controls were performed using non-treated cells exposed to an AMF alone and with cells not exposed to an AMF. The cells were then suspended in media, used to seed 96-well plates at a density of 1.0 × 10^4^ cells/well, and cultured for an additional 48 h. Cell viability was determined using a Calcein AM assay [22].

### 2.8. In Vivo Studies

All animal studies were approved by the Institutional Animal Care and Use Committee of Oregon State University and Oregon Health and Science University (TR01_IP00000033, approval date 29/June/2018).

#### 2.8.1. Development of a Mouse Model of Human Prostate Cancer

DU145 cells (3 × 10^6^) were suspended in Matrigel and subcutaneously injected in the right flank of 4–6-week-old female mice purchased from Charles River Laboratories (Wilmington, MA, USA). Tumor size was measured with a caliper every 48 h, and the volume was calculated as width^2^ × length × 0.5 [27]. The tumors were treated when they reached 50 mm^3^.

#### 2.8.2. Evaluation of Nanocluster Biodistribution

Animals with subcutaneous tumors were administered SiNc-ZnMn-IONCs at a dose of 10 mg of Fe/kg via tail vein injection. The biodistribution of nanoclusters was evaluated with a Pearl^®^ Impulse Small Animal Imaging System at various time points as previously reported [16]. Organs and tumors were collected post euthanasia, and ex vivo tissue imaging was performed by using the same instrument.

#### 2.8.3. Assessment of the Therapeutic Efficacy of Nanocluster-Mediated Hyperthermia

Experiments were performed on nude mice bearing subcutaneous DU145 xenografts. When the tumor volume became ~50 mm^3^, the mice were randomly divided into six groups (five mice per group): (1) no treatment (5% dextrose), (2) AMF (5% dextrose), (3) IONCs, (4) IONCs + AMF, (5) ZnMn-IONCs and (6) ZnMn-IONCs + AMF. To assess the therapeutic efficacy of magnetic hyperthermia, the mice were injected intravenously with nanoclusters (ZnMn-IONCs or IONCs, 10 mg of Fe/kg). At 18 h post-administration, the mice in Groups 2, 4 and 6 were treated with an AMF (420 kHz, 26.9 kA/m) for 30 min. To perform magnetic hyperthermia treatment, the mice were anesthetized using isoflurane in an induction chamber and maintained under anesthesia during treatment by isoflurane administration via a nose cone. Each mouse was positioned inside the induction coil such that only the posterior half of the mouse, including a flank tumor, was exposed to the AMF. Our magnetic hyperthermia system (MSI automation, Wichita, KS) comprises a power supply for delivering an alternating current to the portable heat station that is equipped with a round copper coil. The water jacket inside the copper coil was maintained at 37 °C. The intratumoral temperature was measured using a fiber-optic thermal probe inserted into the center of the tumor, as previously reported [16,28]. Each group was treated once weekly for four weeks using appropriate formulations.

### 2.9. Statistical Analysis

The data were analyzed using descriptive statistics and are presented as mean value ± standard deviation (SD) from 3–5 separate measurements. Comparisons among groups were executed using independent-samples Student’s *t*-tests. Differences between groups were considered statistically significant at *p* < 0.05.

## 3. Results and Discussion

### 3.1. Preparation and Characterization of Nanoclusters

**Figure 1:**
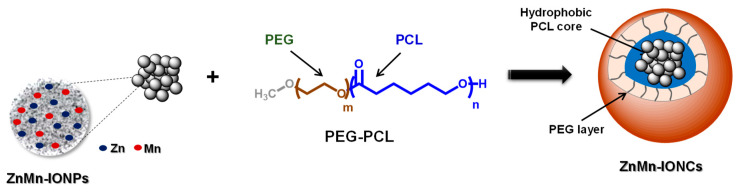
Scheme for nanocluster preparation. To prepare nanoclusters, iron oxide nanoparticles doped with Zn and Mn (ZnMn-IONPs) were encapsulated into the hydrophobic core of a PEG-PCL-based nanocarrier.

The heating efficiency of iron oxide-based nanoparticles in the presence of an AMF relies on their various physico-chemical parameters, including chemical composition. Multiple studies confirmed that the incorporation of some metal dopants (e.g., Zn, Mn, Co, etc.) into the structure of iron oxide nanoparticles, up to certain amounts, can substantially enhance their specific absorption rate (SAR), a measure of nanoparticle heating efficiency [16,29,30,31,32,33]. In addition to their being biocompatible, Zn and Mn were chosen as dopants to enable the tuning of the magnetic characteristics of the nanoparticles in order to improve their specific absorption rate. Previous studies have shown nanoparticles doped with Zn and Mn have higher saturation magnetization, which, in turn, leads to a higher SAR than normal ferrites [34]. Therefore, we developed iron oxide nanoparticles doped with Zn and Mn (ZnMn-IONPs, Figure 1) by using a modified thermal decomposition method.

**Figure 2:**
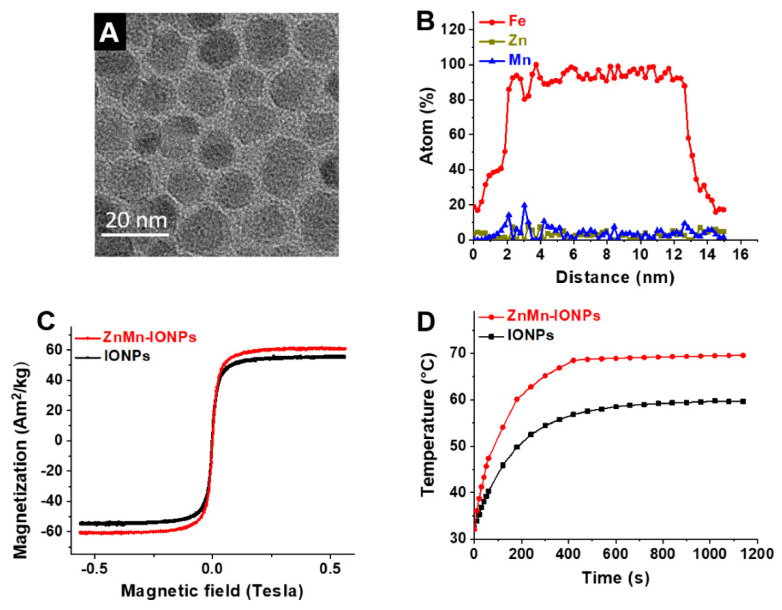
(**A**) Representative TEM image of Zn- and Mn-doped iron oxide nanoparticles (ZnMn-IONPs). (**B**) EDX line scanning profiles of one ZnMn-IONP. (**C**) Magnetization loops of ZnMn-IONPs (red) and IONPs (black) at 300 K. (**D**) Heating curves of ZnMn-IONPs and IONPs (1 mg of Fe/mL in THF) in the presence of an alternating magnetic field (AMF) (420 kHz, 26.9 kA/m).

To prevent the aggregation of nanoparticles, their surface was modified with hydrophobic surfactants, oleic acid and oleylamine. Transmission electron microscopy measurements revealed that nanoparticles with a diameter of 13.32 ± 4.11 nm were synthesized (Figure 2A). Energy dispersive X-ray spectroscopy (EDX) mapping analysis further suggested that each nanoparticle contained 4.07% ± 0.89% Mn and 3.30% ± 0.98% Zn and that these elements are evenly distributed throughout the nanoparticle (Figure 2B).

To assess the improvement in the magnetic properties and heating efficiency of the prepared ZnMn-IONPs, Zn- and Mn-free iron oxide nanoparticles (IONP) with a size of 13.97 ± 3.63 nm were synthesized according to our previously reported procedure and used as a reference [16]. The recorded hysteresis loops (Figure 2C) at room temperature (300 K) demonstrate that ZnMn-IONPs are characterized by a higher saturation magnetization value (*M_s_* = 60.33 Am^2^/kg) than IONPs (57 Am^2^/kg). The hysteresis loops also showed almost no coercivity for both nanoparticles, confirming their superparamagnetic properties at room temperature. In general, nanoparticles with higher saturation magnetization values demonstrate enhanced heating efficiency upon exposure to an AMF [16,29,31]. Our heating measurements confirm (Figure 2D) that ZnMn-IONPs dispersed in THF have a significantly higher SAR value (1615.1 W/g) when compared to IONPs (586.2 W/g) in the presence of an AMF (420 kHz; 26.9 kA/m). The heating efficiency of the developed ZnMn-IONPs is also comparable to that of our previously reported Co- and Mn-doped iron oxide nanoparticles (1718.0 W/g) [16].

**Figure 3:**
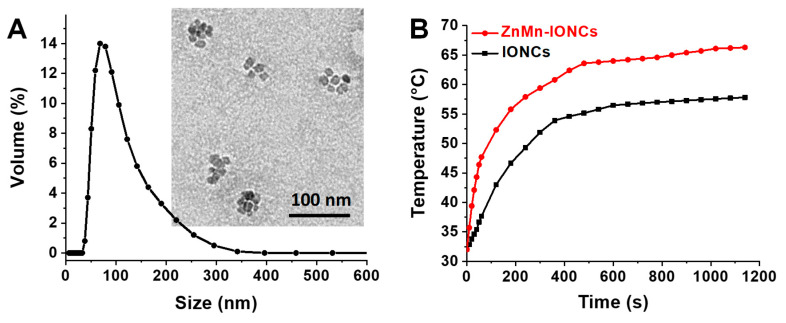
(**A**) Size distribution of ZnMn-IONCs tested by dynamic light scattering. Inset: representative TEM image of ZnMn-IONCs. (**B**) Heating curves of ZnMn-IONCs and IONCs (1 mg of Fe/mL in water) exposed to AMF (420 kHz, 26.9 kA/m).

To obtain water-soluble nanoparticles for systemic delivery, the prepared hydrophobic ZnMn-IONPs were encapsulated into the core of PEG-PCL-based polymeric nanoparticles following our previously reported solvent evaporation method (Figure 1) [16,27,28]. The selected PEG-PCL nanoparticles possess many attractive features as delivery vehicles, including biocompatibility and the ability to encapsulate various hydrophobic nanomaterials and efficiently transfer them to cancer tumors following systemic administration [16,27,28]. TEM imaging revealed that multiple ZnMn-IONPs were clustered inside the PEG-PCL nanoparticles (Figure 3A). It was reported that clusters of nanoparticles are characterized by a higher heating efficiency when compared to their individual counterparts under the same experimental conditions [16,29,35]. The average hydrodynamic size and surface charge of the prepared nanoclusters (ZnMn-IONCs) were 109.4 ± 0.65 nm (Figure 3A) and −1.96 ± 0.43 mV, respectively.

The heating efficiency study shows that ZnMn-IONCs rapidly increased the aqueous solution temperature above 60 °C, while the solution temperature of the IONCs was 10 degrees less under the same conditions (Figure 3B). Finally, the calculated SAR value of the ZnMn-IONCs (1010.0 W/g) was two times higher than the SAR value of the IONP nanocluster (IONCs, 493.8 W/g) and comparable with the heating efficiency of our previously reported nanoclusters of Co- and Mn-doped IONPs (1237.0 W/g) exposed to the same AMF (420 kHz; 26.9 kA/m) [16]. The decrease in the SAR value of ZnMn-IONCs (1010.0 W/g) in aqueous solution when compared to the SAR of ZnMn-IONPs in THF (1615.1 W/g) is related to the fact that measurements of heating efficiency were performed in solvents with different viscosities. The obtained results are in good agreement with our previous report suggesting that individual nanoparticles in THF with lower viscosity (0.48 cP at 25 °C) demonstrate significantly higher heating efficiency than the same nanoparticles transferred to water with higher viscosity (0.89 cP at 25 °C) [16]. Jang et al. reported that nanoparticles demonstrate lower heating efficiency in a solvent with a higher viscosity due to the reduction of “Brownian relaxation loss power caused by the increase of Brownian relaxation time” [33].

### 3.2. In Vitro Evaluation of Nanoclusters

**Figure 4:**
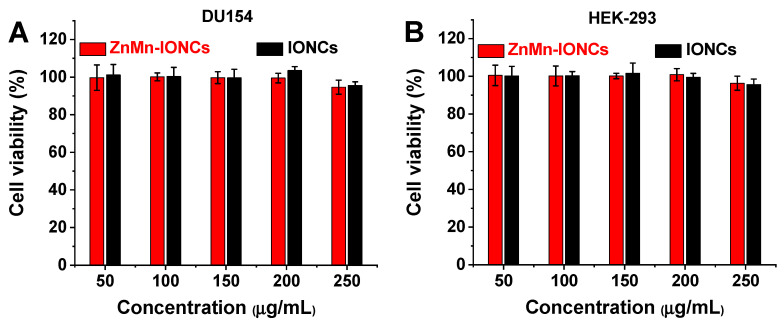
Viability of human prostate cancer DU145 cells (**A**) and human embryonic kidney HEK-293 cells (**B**) treated for 24 h with various concentrations of ZnMn-IONCs and IONCs (50–250 μg of Fe/mL).

Magnetic hyperthermia is a targeted therapy aimed at killing cancer cells with an elevated temperature generated by non-toxic nanoparticles in the presence of a safe AMF. The safety and biocompatibility of the AMF (420 kHz, 26.9 kA/m) employed in the current study were extensively evaluated both in vitro and in vivo and described in our previous report [16]. To assess the potential toxicity of our nanoclusters alone, both human prostate cancer cells (DU145) and non-malignant human embryonic kidney cells (HEK-293) were incubated with various concentrations of ZnMn-IONCs and IONCs (50–250 μg of Fe/mL) for 24 h. The obtained results confirm that ZnMn-IONCs and IONCs at the tested concentrations do not show any significant toxicity to either cancer or non-malignant cells (Figure 4A,B).

**Figure 5:**
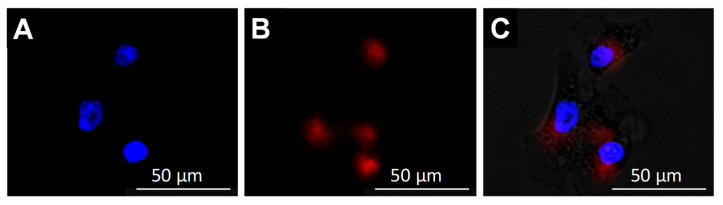
Fluorescence microscopy images of DU145 cells demonstrating intracellular localization of ZnMn-IONCs loaded with a near-infrared (NIR) dye, SiNc: (**A**) DAPI staining, (**B**) SiNc-ZnMn-IONCs and (**C**) overlay.

To investigate the cellular internalization and intracellular distribution of the developed nanoclusters, DU145 prostate cancer cells were incubated with ZnMn-IONCs loaded with a near-infrared (NIR) dye, SiNc, for 24 h. Fluorescence microscopy imaging verified that a strong NIR fluorescence signal generated by the hydrophobic dye encapsulated inside the ZnMn-IONCs was present in the cytoplasm of the cancer cells following incubation with nanoclusters (Figure 5).

**Figure 6:**
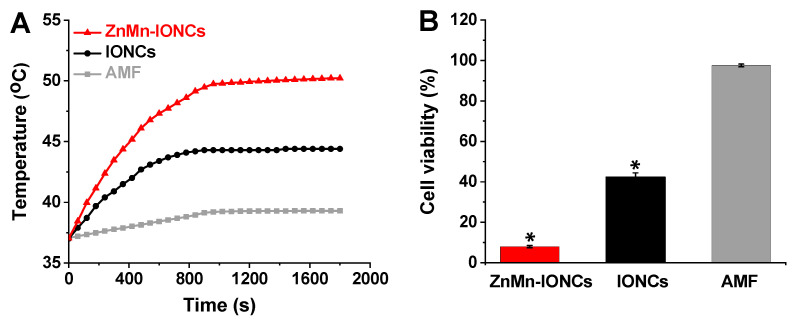
(**A**) Representative heating curves of DU145 cells treated with medium only (AMF), ZnMn-IONCs (50 μg of Fe/mL) and IONCs (50 μg of Fe/mL) for 24 h, and exposed to AMF (420 kHz, 26.9 kA/m). (**B**) The viability of DU145 cells treated with ZnMn-IONCs (50 μg of Fe/mL), IONCs (50 μg of Fe/mL) and medium (AMF) for 24 h and subjected to AMF for 30 min. * *p* < 0.05 when compared with untreated cells.

Finally, to assess the cellular heat generation efficiency and anti-cancer effect of both ZnMn-IONCs and IONCs, DU145 cells were treated with nanoclusters (50 μg of Fe/mL) for 24 h, and cell pellets were subjected to an AMF (420 kHz, 26.9 kA/m) for 30 min. The temperature of the cells treated with ZnMn-IONCs reached 50 °C in 15 min and remained constant during the AMF exposure (Figure 6A, red curve). In the case of IONCs, the cellular temperature did not rise above 44 °C under identical experimental conditions (Figure 6A, black curve). The cell viability measurements reveal that more than 90% cancer cell death is achieved with ZnMn-IONC-mediated magnetic hyperthermia, while the treatment mediated by IONCs reduced cell viability by only ~60% under the same experimental conditions (Figure 6B). The obtained results also confirm that an AMF alone increases cellular temperature by only ~2 °C (Figure 6A, gray curve), and therefore, it does not compromise the viability of the tested prostate cancer cells (Figure 6B).

### 3.3. In Vivo Evaluation of Nanoclusters

**Figure 7:**
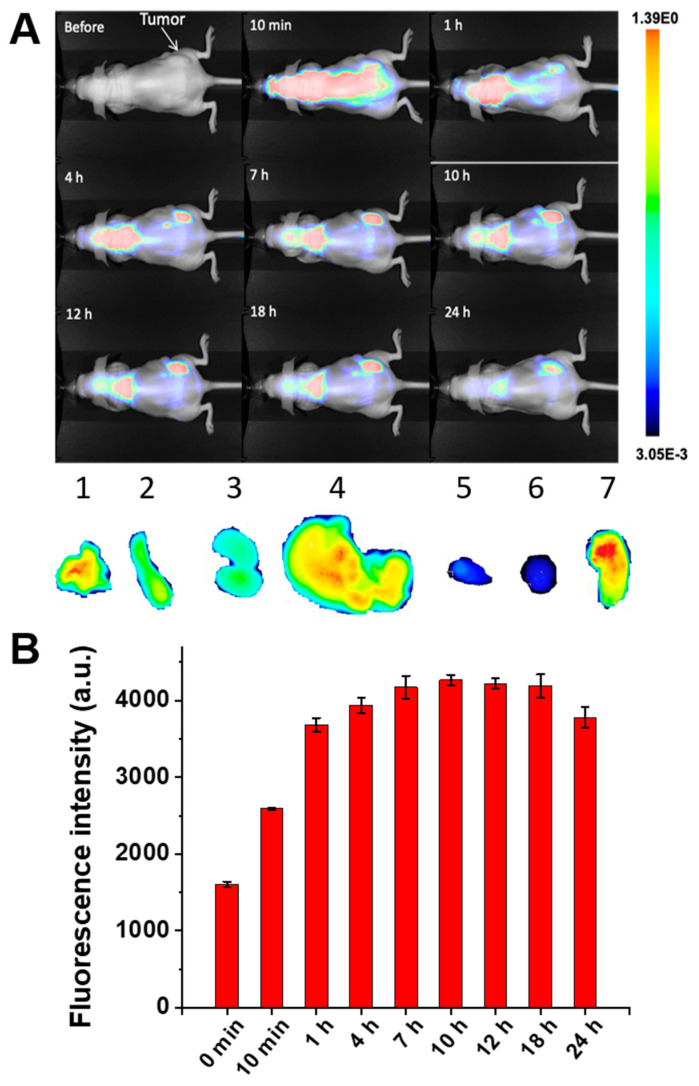
(**A**) NIR fluorescence images of a mouse with DU145 xenograft at different time points following intravenous administration of SiNc-loaded ZnMn-IONCs (top panel). Organs and tumor distribution of SiNc-loaded ZnMn-IONCs 18 h after intravenous injection (lower panel). 1—lungs; 2—spleen; 3—kidneys; 4—liver; 5—heart; 6—brain; 7—tumor. (**B**) The NIR fluorescence intensity was measured in the tumor as a function of time after intravenous injection of SiNc-ZnMn-IONCs in a mouse bearing a DU145 tumor.

Based on the promising in vitro results, we further evaluated the safety and therapeutic efficacy of the systemically delivered magnetic hyperthermia mediated by our nanoclusters in nude mice bearing subcutaneous xenografts of DU145 prostate cancer cells. The main prerequisite for systemically delivered magnetic hyperthermia is the ability of the nanoparticles to efficiently reach cancer tumors after intravenous administration and raise intratumoral temperature above 42 °C in the presence of the external AMF. The whole body fluorescence imaging of mice at various time points and the following semi-quantitative analysis of the fluorescence intensity in the tumor area revealed that SiNc-loaded ZnMn-IONCs started to accumulate in cancer tumors via passive targeting within 1 h post-injection, and reached a maximum accumulation at 7 h (Figure 7A,B). The fluorescence intensity in the tumor area started to decrease at 24 h, suggesting that the optimal time window for the exposure of cancer tumors to the external AMF is between 7 and 24 h after the intravenous injection of the developed ZnMn-IONCs. The ex vivo imaging of the resected tissues at 18 h post-injection revealed a higher fluorescence intensity in the tumor when compared to the liver, kidneys, spleen and lungs (Figure 7A). The obtained results are in good agreement with previous reports demonstrating that, in addition to cancer tumors, PEG-PCL nanoparticles can accumulate in the above-mentioned organs following systemic administration [27,36,37]. It is a known fact that, regardless of their size, charge, surface modification and other parameters, nanoparticles face various biological barriers in the body, including the reticuloendothelial system [38]. Therefore, a portion of the injected nanoparticles accumulates in the liver, spleen, lungs or other organs. The major goal of effective nanoparticle design for magnetic hyperthermia is to maximize nanoparticle delivery to cancer tumors via passive or active targeting [39] while minimizing their accumulation in healthy tissues [38]. Therefore, to avoid collateral damage to healthy tissues during magnetic hyperthermia, an AMF should be applied at the optimal time after the injection of nanoparticles when the maximum accumulation of nanoclusters in tumors and maximum clearance from healthy tissue are achieved. Furthermore, the focused AMF can be delivered to cancer sites, preventing the potential heating of healthy tissues containing nanoparticles. For example, Magnetic Insight, Inc. recently developed the HYPER system, which offers millimeter-scale spatial control of magnetic heating. By using this technology, Tay et al. demonstrated the localization of magnetic hyperthermia to cancer tumors while minimizing damage to the nearby liver (1–2 cm distance) [40]. In our studies, only the posterior half of the mouse, including a flank tumor, was exposed to the AMF.

**Figure 8:**
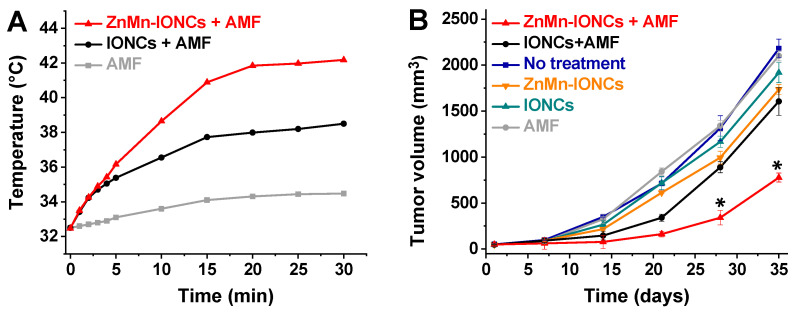
(**A**) Intratumoral temperature curves during AMF (420 kHz, 26.9 kA/m) exposure of animals treated with 5% dextrose (AMF), ZnMn-IONCs (10 mg of Fe/kg) and IONCs (10 mg of Fe/kg). (**B**) Tumor growth profiles after four cycles of the following treatments: (i) no treatment; (ii) ZnMn-IONCs + AMF, mice injected with ZnMn-IONCs (10 g of Fe/kg) and subjected to AMF for 30 min; (iii) IONCs + AMF, mice injected with IONCs (10 g of Fe/kg) and subjected to AMF for 30 min; (iv) ZnMn-IONCs, mice injected with ZnMn-IONCs (10 g of Fe/kg); (v) IONCs, mice injected with IONCs (10 g of Fe/kg); and (vi) AMF, mice injected with 5% dextrose and exposed to AMF. * *p* < 0.05 when compared with non-treated animals.

To assess the efficiency of the developed nanoclusters in elevating the intratumoral temperature, mice were intravenously injected with ZnMn-IONCs at a dose of 10 mg of Fe/kg and exposed to an AMF (420 kHz, 26.9 kA/m) at 18 h post-injection. The temperature profiles recorded with a fiber-optic thermal probe demonstrate that ZnMn-IONCs can elevate the intratumoral temperature above 42 °C in the presence of an AMF, while tumors treated with IONCs reached only 38.5 °C under the same conditions (Figure 8A). Finally, we confirmed that the employed AMF alone is safe and only negligibly increases the intratumoral temperature (<2 °C) due to the generation of eddy currents. We have not evaluated temperature in various organs upon AMF exposure because it is challenging to perform these measurements in live mice with a fiber-optic thermal probe.

To assess the therapeutic efficacy of systemically delivered magnetic hyperthermia for prostate cancer treatment; mice were intravenously injected with the developed nanoclusters and exposed to an AMF for 30 min once per week for four weeks. The obtained results revealed that four cycles of magnetic hyperthermia significantly inhibited the growth of prostate cancer tumors when compared to IONC-mediated therapy (Figure 8B). The tumor volumes of the mice treated with ZnMn-IONC-mediated hyperthermia were ~two times smaller than those of the tumors exposed to IONC-mediated treatment. Of note, nanoparticles and the AMF alone did not show a significant effect on tumor growth (Figure 8B).

**Figure 9:**
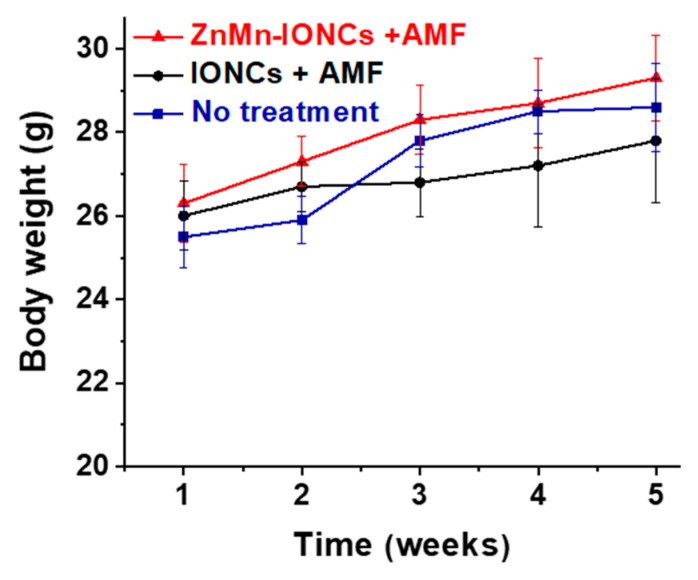
Changes in body weights of non-treated mice and animals exposed to 4 cycles of ZnMn-IONC- and IONC-mediated hyperthermia.

The animals were monitored for two weeks following the treatment for toxicity evaluation. There were no signs of toxicity (e.g., appearance or death) or weight loss in the mice treated with the proposed ZnMn-IONC-mediated hyperthermia (Figure 9).

## 4. Conclusions

In summary, we have successfully developed novel nanoclusters of zinc- and manganese-doped iron oxide nanoparticles encapsulated inside PEG-PCL-based polymeric vehicles. The developed nanoclusters demonstrate comparable heating efficiency to our previously reported nanoclusters of cobalt- and manganese-doped iron oxide nanoparticles, but without the putative toxicity associated with cobalt. We observed no acute toxicity in mice after four injections with the developed nanoclusters and following exposure to an AMF. The experimental findings further confirm that our nanoclusters efficiently accumulate in prostate cancer xenografts via passive targeting within seven hours following intravenous injection, and generate the required therapeutic temperature inside tumors upon exposure to a safe AMF. Finally, we demonstrate for the first time that systemically delivered magnetic hyperthermia is a feasible treatment modality for prostate cancer and that it can efficiently inhibit tumor growth without causing any obvious toxicity in mice. Further studies are planned to confirm the long-term safety of the developed nanoclusters and investigate the therapeutic outcomes of the proposed magnetic hyperthermia for prostate cancer treatment in combination with other anti-cancer therapies, including chemotherapy.
